# Supplementary material for: Identification and Characterization of RK22, a Novel Antimicrobial Peptide from Hirudinaria manillensis against Methicillin Resistant Staphylococcus aureus
Source: Int J Mol Sci. 2023 Aug 30;24(17):13453. doi: 10.3390/ijms241713453 (PMC10487658; doi:10.3390/ijms241713453)
Supplement: Supplementary file 1 [file ijms-24-13453-s001.zip › ijms-2547062-supplementary.pdf]

Supplementary materials

Supplementary Tables and Tables legends

Table S1. The stability of RK22 in plasma within 10 h.

time (h) / Bacteria strain	0	2	4	6	8	10
S. aureus (ATCC6538)	6.25	6.25	6.25	6.25	6.25	6.25
MRSA-Z	6.25	6.25	6.25	6.25	6.25	6.25

Supplementary Figures and Figure legends

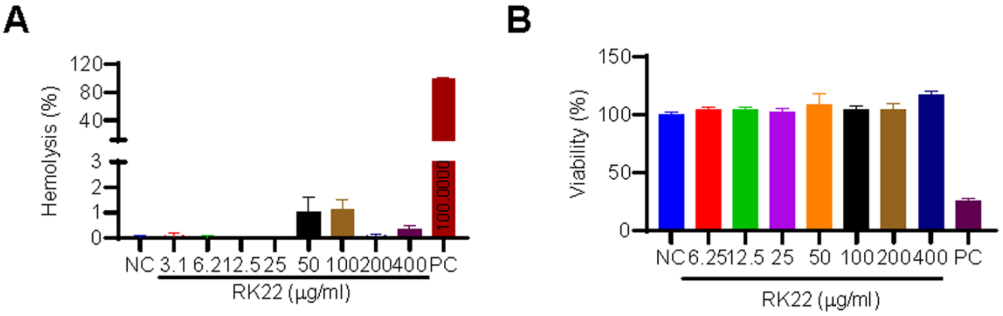

Figure S1. Effects of RK22 on Hemolysis and cytotoxicity. 100 μl mouse blood cell suspension was incubated with 100 μl of RK22 at different concentrations (0–400 μg/mL). After incubation for 60 min at 37°C, cells were centrifuged (3,500 rpm, 5 min) and the absorbance of the supernatant was measured at 540 nm. The value for "zero hemolysis" was determined using sterile saline (negative control, NC), while 100% hemolysis was established using 1% (v/v) Triton X-100 (PC: positive control). Hemolysis of testing sample was calculated as the percentage of Triton X-100-induced hemolysis. (B) HEK293T cells (2×10^4 cells) were plated into 96 well plates and incubated with RK22 (0–400 μg/mL) or 10% DMSO (PC: positive control) for 24 h. Cell viability was determined by adding 10 μl CCK8 in to the cells and incubated for 1-4 h. The absorbance at 450 nm of the solution was measured with a microplate reader. Data represent mean ± SD of 3 independent experiments.

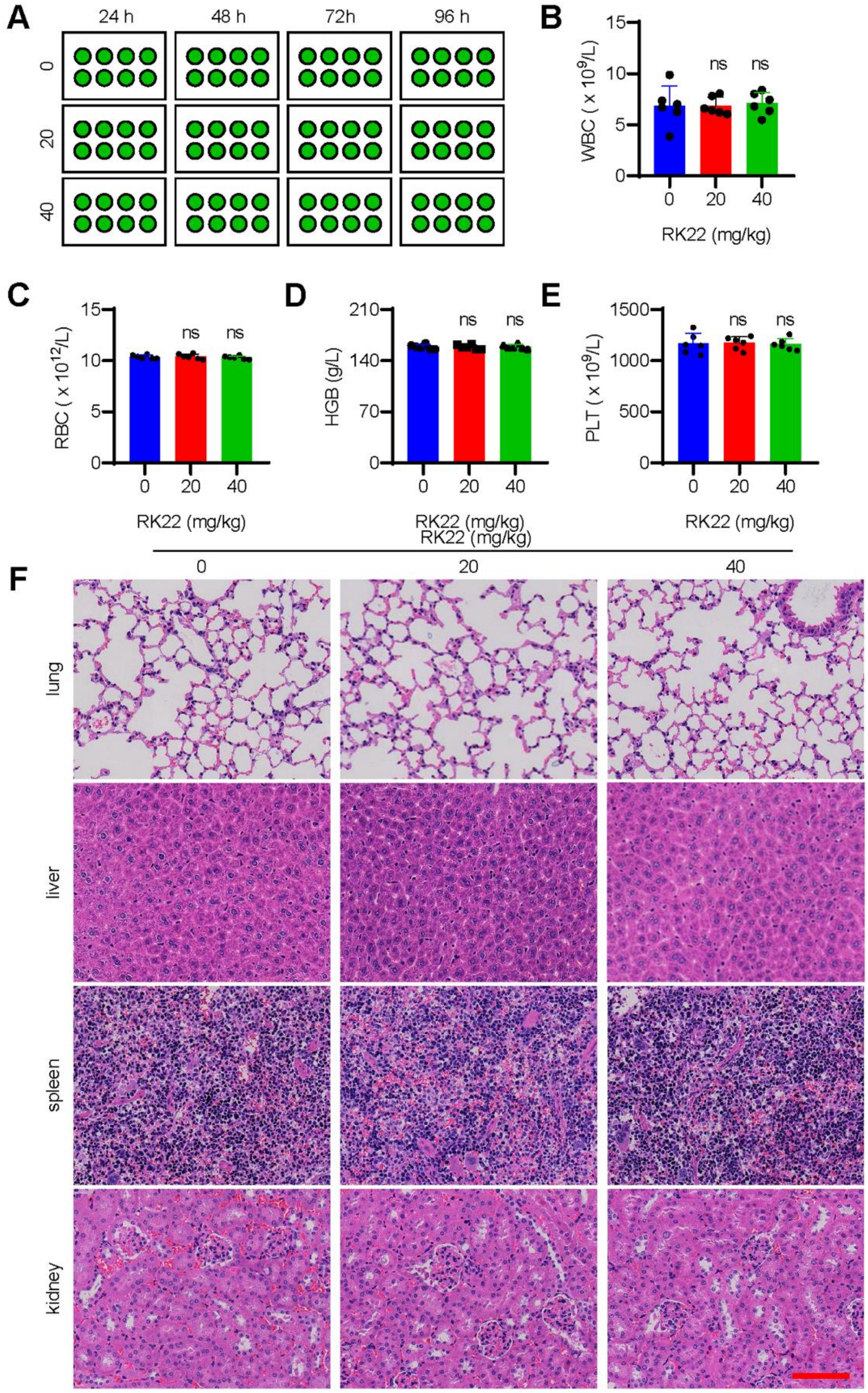

Figure S2. ***In vivo*** **acute toxicity of RK22.** Male C57BL/6 mice (6–8 weeks of age, n = 8 /group) were injected intravenously via the tail vein with saline or RK22 (20 and 40 mg/kg). Mice were observed for survival status within 96 h of the injection (A) and blood was collected via retro-orbital bleeding for routine blood analysis (B-E). The sections of lung, liver, spleen and kidney were stained

with hematoxylin & eosin (H&E) for histopathological analysis, scale bar:100 μm. Data represent mean ± SD of 3 independent experiments.
